# Supplementary material for: A class-mismatched TCR bypasses MHC restriction via an unorthodox but fully functional binding geometry
Source: Nat Commun. 2022 Nov 23;13:7189. doi: 10.1038/s41467-022-34896-0 (PMC9691722; doi:10.1038/s41467-022-34896-0)
Supplement: Supplementary file 1 — Supplementary Information [file 41467_2022_34896_MOESM1_ESM.pdf]

Supplementary Information

A class-mismatched TCR bypasses MHC restriction via an unorthodox but fully functional binding geometry

Nishant K. Singh, Jesus A. Alonso, Jason R. Devlin, Grant L. J. Keller, George I. Gray, Adarsh K. Chiranjivi, Sara G. Foote, Lauren M. Landau, Alyssa G. Arbuiso, Laura I. Weiss, Aaron M. Rosenberg, Lance M. Hellman, Michael I. Nishimura, and Brian M. Baker

Supplementary Figure 1: Interatomic TCR-peptide/MHC contacts.

Supplementary Figure 2: Effects of mutations in the TIL1383I CDR3β and CDR3α loops.

Supplementary Figure 3: Effects of mutation of Arg65 of HLA-A2 to alanine.

Supplementary Figure 4: Structural alignment of class II MHC onto class I and comparison of key residues.

Supplementary Figure 5: Overview of the rapid process for K_D determination using the UV-exchanged peptide/HLA-A2 library.

Supplementary Figure 6: Differential scanning fluorimetry analysis of the UV-exchanged peptide/HLA-A2 library.

Supplementary Figure 7: Flow cytometry gating.

Supplementary Table 1: X-ray data and refinement statistics.

Supplementary Table 2: Oligonucleotides used for site directed mutagenesis.

a

		TCR α chain										TCR β chain									
		1α			2α		3α					1β	2β	3β							
		THR	ASN	TYR	TYR	LYS	ASN	TYR	GLY	GLY	GLN	ARG	ASP	THR	GLU	GLY	LEU	ILE	PHE	PRO	
		36	37	39	58	59	100	101	102	103	105	34	58	100	101	104	105	106	107	108	Total
	GLU	19										2 •									2
HLA-A2 α1 helix	ARG	65							1	3											4
	LYS	66						3	3												6
	LYS	68											•								0
	ALA	69							1												1
	GLN	72												2	2						4
	THR	73																		2	2
	ARG	75										6			6 •						12
	VAL	76												1	1						2
HLA-A2 α2 helix	LYS	146														3	5				8
	ALA	150			5																5
	HIS	151				5	3														8
	GLU	154				1	4 •														5
	GLN	155	3	11	7	3		4													28
	ALA	158	1																		1 TCR:HLA 88
Peptide	TYR	1							3												3
	GLY	4						1	8	2											11
	THR	5		5				2													7
	MET	6						10	3	2		4							2	1	22
	SER	7																2			2
	GLN	8															3	3		1	7 TCR:pep 52
		Total	4	16	12	9	7	17	17	9	3	4	8	0	3	9	3	8	5	2	4

b

DMF4 (PDB 3QEQ) CDR1/2 β chain contacts to HLA-A2

		Shared TCR β chain					
		1β		2β			
		HIS	TYR	TYR	TYR	ASP	ASP
		29	31	48	50	54	56
ARG	65			2	1		
LYS	68						1
ALA	69		4		2		
GLN	72		1		6	1	
ARG	75					•	
VAL	76	5					

38-10 (PDB 6VRN) CDR1/2 β chain contacts to HLA-A2

		Shared TCR β chain					
		1β		2β			
		ARG		TYR	ASP	ASP	
		30		50	54	56	
ARG	75				1 •	1 •	
VAL	76			1			
THR	80	2					
LYS	146	2					

c

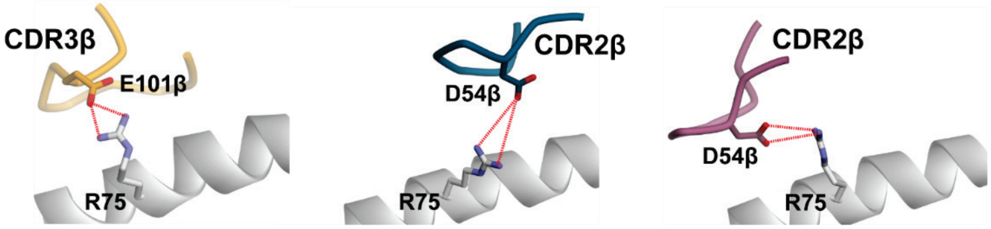

TIL1383I DMF4 38-10

Supplementary Figure 1. Interatomic TCR-peptide/MHC contacts.

a) Contacts between amino acids in the interface between TIL1383I and Tyr_{370D}/HLA-A2. Contacts are tabulated as interatomic distances ≤ 4 Å. The total number of contacts is colored from white (minimum) to purple (maximum). The presence of one or more hydrogen bonds is indicated by a red square; the presence of one or more salt bridges is indicated by a red dot (salt bridge distances can be > 4 Å).

b) Contacts between the CDR1β and CDR2β loops and HLA-A2 in the DMF4 (left) and 38-10 (right) TCRs, which share *TRBV10-3* with TIL1383I, tabulated and colored as in panel **a**.

c) In the CDR2β loops of the *TRBV10-3* TCRs DMF4 and 38-10, Asp54 forms salt bridges with Arg75 on the HLA-A2 α1 helix. In the TIL1383I structure, however, this salt-bridge is formed by Glu101 in CDR3β.

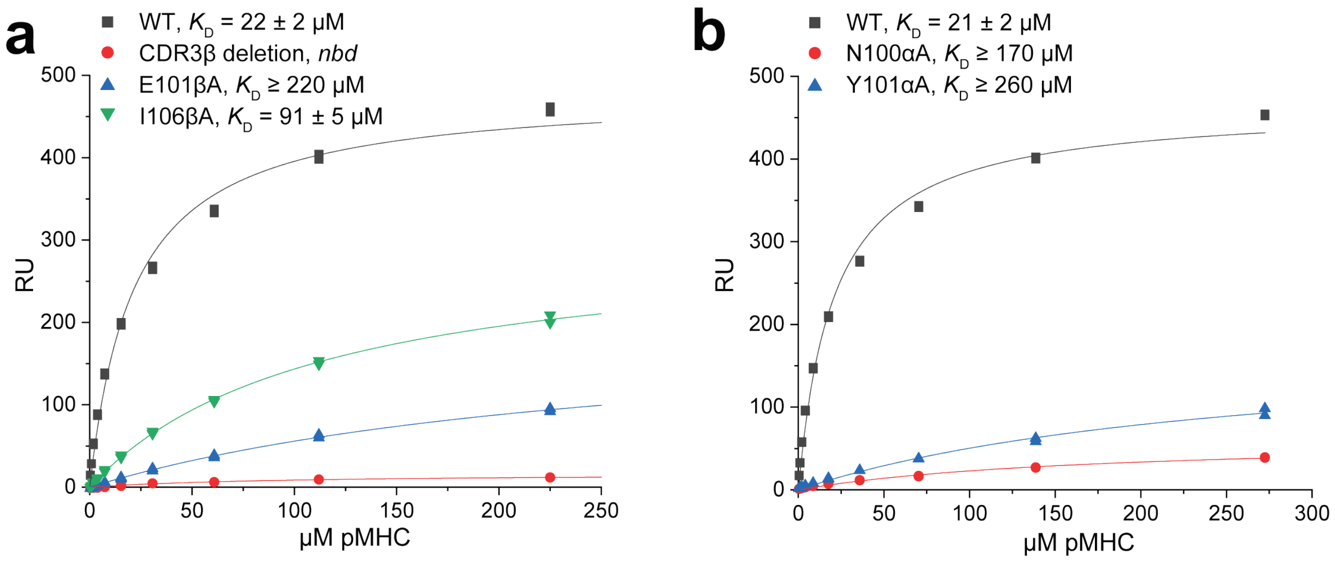

Supplementary Figure 2. Mutations in the TIL1383I CDR3β and CDR3α loops confirm important interactions with Tyr$_{370D}$/HLA-A2 seen in the TCR-pMHC crystal structure as measured by surface plasmon resonance (SPR).

a) Mutations in CDR3β and their effects on TCR binding. CDR3β deletion (red) refers to a mutant in which Glu102, Gly103, and Gly104 of the CDR3β are deleted, shortening the loop by three amino acids. No binding was detected (*nbd*) for this experiment. Mutation of Glu101β (blue) and Ile106β (green) in the CDR3β loop to alanine substantially weaken binding.

b) Mutation of Asn100α (red) and Tyr101α (blue) in the CDR3α loop also substantially weaken binding. Errors in both panels are standard fitting errors from simultaneous analysis of two datasets. Source data are provided in the Source Data file.

a

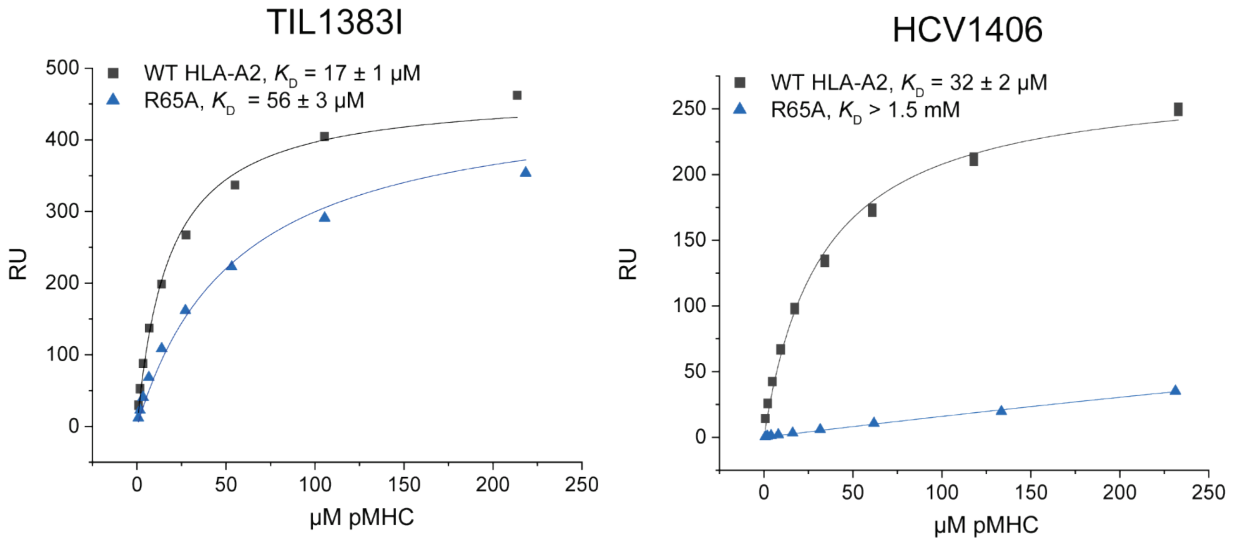

b

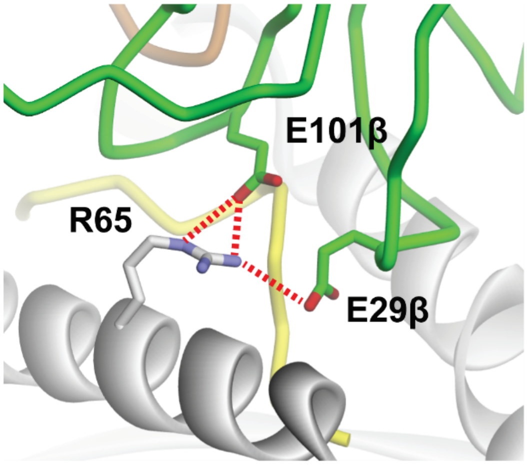

Supplementary Figure 3. Mutation of Arg65 of HLA-A2 to alanine has a large effect on the binding of the canonically binding TCR HCV1406, but a much smaller effect with TIL1383I.

a) SPR titrations with fits to the data and fitted K_D values for TIL1383I binding Tyr_{370D}/HLA-A2 (left) and HCV1406 binding NS3/HLA-A2 (right). The ΔΔG° for TIL1383I is 0.7 kcal/mol, whereas for HCV1406 it is > 2.3 kcal/mol. Errors are standard fitting errors from simultaneous analysis of two datasets. Source data are provided in the Source Data file.

b) Electrostatic interactions involving Arg65 in the HCV1406-NS3/HLA-A2 interface. Compare with Fig. 4a, which shows Arg65 in the TIL1383I interface.

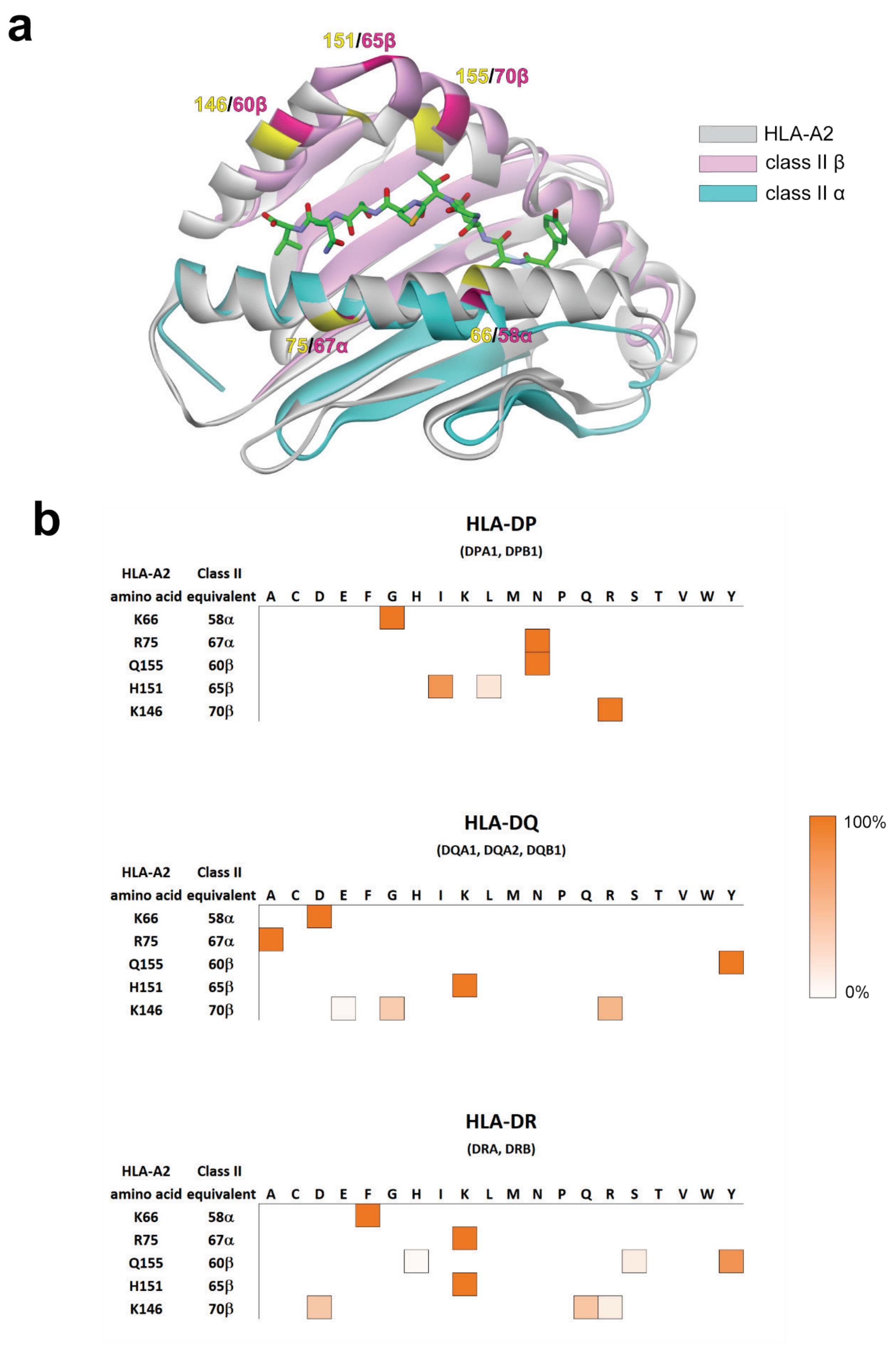

Supplementary Figure 4. Structural alignment of class II MHC onto class I and comparison of key residues.

a) Structural alignment with key positions in HLA-A2 and their equivalents in the class II α1 and β1 helices highlighted.

b) Amino acids found at the positions highlighted in panel **a** in HLA-DP (top), DQ (middle), and DR genes. Percentage of each amino acid at each position are indicated via the white-orange scale at the right.

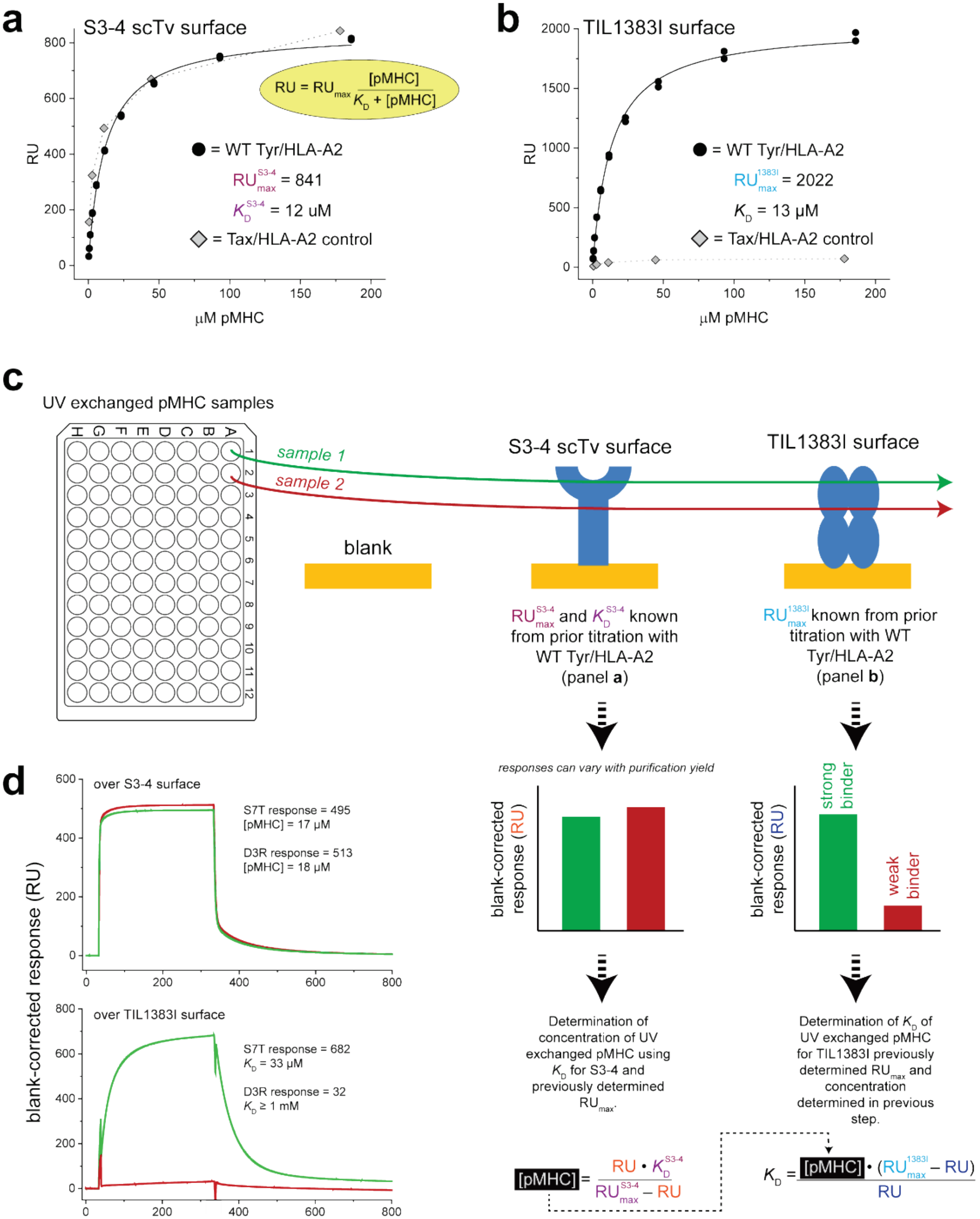
a
S3-4 scTv surface
RU = RUmax [pMHC] / (KD + [pMHC])
● = WT Tyr/HLA-A2
RUmax S3-4 = 841
KD S3-4 = 12 uM
◇ = Tax/HLA-A2 control
RU
µM pMHC
b
TIL1383I surface
● = WT Tyr/HLA-A2
RUmax 1383I = 2022
KD = 13 µM
◇ = Tax/HLA-A2 control
RU
µM pMHC
c
UV exchanged pMHC samples
sample 1
sample 2
blank
S3-4 scTv surface
TIL1383I surface
RUmax S3-4 and KD S3-4 known from prior titration with WT Tyr/HLA-A2 (panel a)
RUmax 1383I known from prior titration with WT Tyr/HLA-A2 (panel b)
responses can vary with purification yield
blank-corrected response (RU)
strong binder
weak binder
Determination of concentration of UV exchanged pMHC using KD for S3-4 and previously determined RUmax.
Determination of KD of UV exchanged pMHC for TIL1383I previously determined RUmax and concentration determined in previous step.
[pMHC] = RU • KD S3-4 / (RUmax S3-4 – RU)
KD = [pMHC] • (RUmax 1383I – RU) / RU
d
over S3-4 surface
S7T response = 495
[pMHC] = 17 µM
D3R response = 513
[pMHC] = 18 µM
over TIL1383I surface
S7T response = 682
KD = 33 µM
D3R response = 32
KD ≥ 1 mM
blank-corrected response (RU)

Supplementary Figure 5 (previous page). Overview of the process for K_D determination using the UV-exchanged peptide/HLA-A2 library.

a, b) After coupling to adjacent SPR sensor surfaces, the affinity (K_D) and active surface densities (RU_{max}) of the S3-4 scTv (panel **a**) and TIL1383I TCR (panel **b**) were determined through traditional SPR steady state titrations, shown here for representative titrations. Tax/HLA-A2 is an irrelevant peptide/HLA-A2 complex (peptide sequence LLFGYPVYV) that is recognized by S3-4 but not TIL1383I. The equation circled in yellow describes simple 1:1 binding; this equation is manipulated for relevant variables as shown at the bottom of panel **c**. Parameters from the fitted curves are colored to highlight mathematical manipulations. Source data are provided in the Source Data file.

c) Following determination of the K_D and RU_{max} for the S3-4 and TIL1383I surface, samples from the UV-exchanged peptide/HLA-A2 library were injected sequentially over the S3-4 and TIL1383I surface (along with a blank surface for bulk response correction). The blank-corrected response (RU) from the scTv surface, together with the previously determined K_D and RU_{max} for the S3-4 surface, allows calculation of the active concentration of peptide/HLA-A2. The blank-corrected response from the TIL1383I surface, together with the active concentration of peptide/HLA-A2 and previously determined RU_{max} for the TIL1383I surface, allows determination of the K_D. Red and green bars illustrate responses for peptide/HLA-A2 samples that are recognized similarly by S3-4 as expected (but with SPR responses that vary with exchange/purification yield), but are recognized either strongly or weakly by TIL1383I.

d) Representative data for strong and weak TIL1383I binders as diagrammed in panel **c** (Tyr_{370D} pS7→T and pD3→R). Both samples gave good responses over the S3-4 surface as expected, yielding concentrations of 17-18 μM. The strong binder gives a good response over the TIL1383I surface, indicating an affinity of 33 μM for pS7→T, whereas the affinity of the weak binder pD3→R is estimated to be ≥ 1 mM. Source data are provided in the Source Data file.

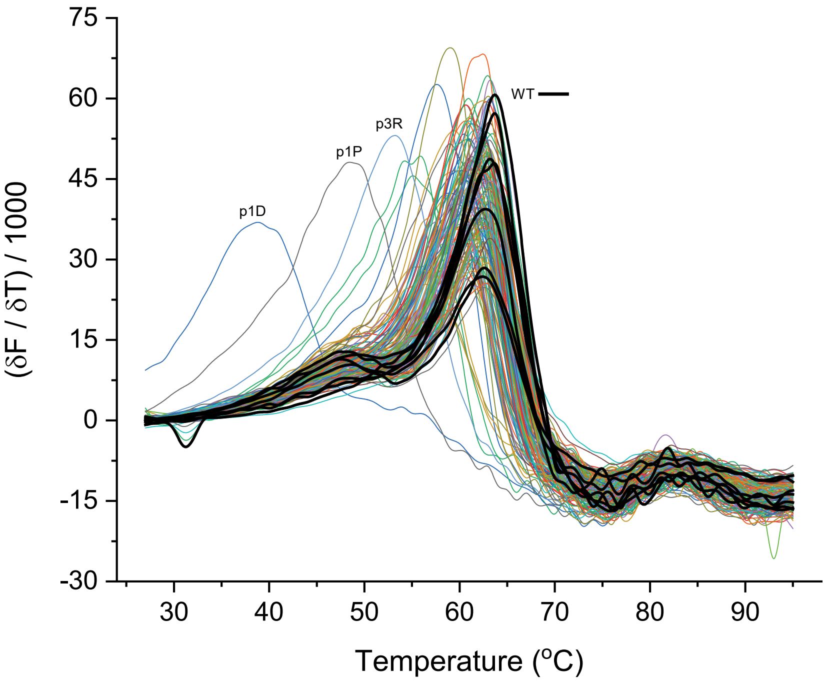

Supplementary Figure 6. Differential scanning fluorimetry analysis of the UV-exchanged peptide/HLA-A2 library, plotted as the first derivative of the fluorescence response with respect to temperature. Solid black lines are the seven UV-exchanged WT Tyr_{370D}/HLA-A2 complexes, all showing T_m values of 62-63 °C. Melting curves for complexes with substantially weaker T_m values are highlighted (p1D, p1P, p3R). Source data are provided in the Source Data file.

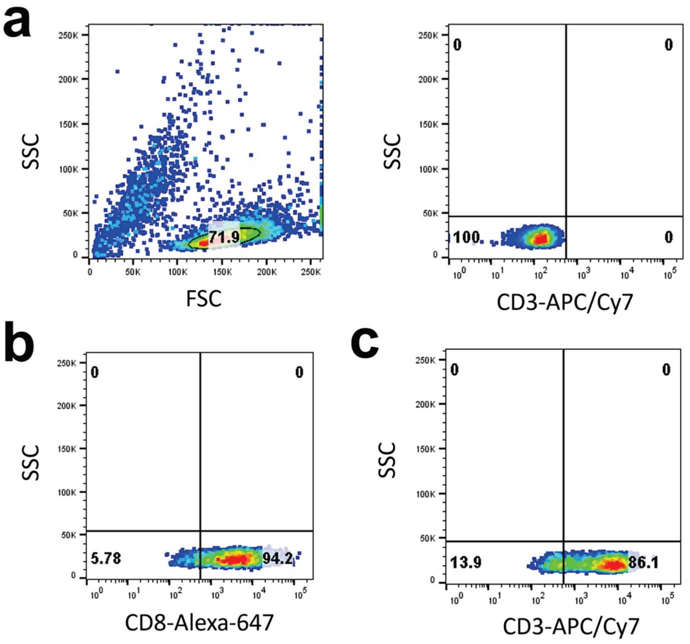

Supplementary Figure 7. Flow cytometry gating.

a) Analysis of untransduced Jurkat 76 cells. Left panel shows side scatter (SSC) vs. forward scatter (FSC) gating strategy for identifying viable single cells as defined by the “singlets” gate, with percent of that subpopulation shown. Right panel shows singlet-gated subpopulations analyzed for CD3 expression by anti-human CD3-APC/Cy7 antibody confirming lack of TCR.

b, c) Analysis of TIl1383I expressing CD8+ Jurkat 76 cells. Panel **b** shows side scatter (SSC) vs. CD8-Alexa 647 staining to demonstrate levels of CD8 expression. Panel **c** shows cells transduced with full-length TIl1383I TCR, tested for CD3 expression by anti-human CD3-APC/Cy7 antibody, confirming the presence of TCR.

Supplementary Table 1. X-ray data and refinement statistics.

Data collection	
Resolution (Å)*	36.31 - 2.54 (2.63 - 2.54)
Space group	C 1 2 1
Unit cell dimensions (Å)	114.74, 74.44, 131.06
Unit cell angles (°)	90, 93.49, 90
Unique reflections*	36,440 (3,527)
Multiplicity*	1.0 (1.0)
Completeness (%)*	99.52 (96.87)
Mean I/σ*	8.27 (2.07)
R_{meas}*	0.16 (1.05)
R_{pim}*	0.06 (0.43)
$CC_{1/2}$*	0.99 (0.69)
Refinement	
R_{work}	0.21
R_{free}	0.25
Reflections used in refinement*	36,430 (3,525)
Reflections used for R_{free}*	2,006 (201)
Wilson B-factor (Å^2)	56.46
Number of non-hydrogen atoms	6,295
macromolecules	6,213
ligands	0
solvent	82
Protein residues	773
Bond lengths RMSD (Å)	0.005
Average B-factor (Å^2)	89.5
TCR	68.5
peptide	72.4
MHC	117.6
Bond angles RMSD (°)	0.92
Ramachandran favored (%)	97.51
Ramachandran allowed (%)	2.49
Ramachandran outliers (%)	0
Rotamer outliers (%)	1.32
Clashscore	8.85
PDB ID Code	7RK7

* Statistics for the highest-resolution shell are shown in parentheses.

Supplementary Table 2. Oligonucleotides used for site directed mutagenesis.

Primer Name	Sequence	Description
TIL1383I-N100αA-FP	GGTGGCCCTGGCCTATGGCGGTAGC	Asn 100 to Ala in α chain forward
TIL1383I-N100αA-RP	AGGCAGTAATAAACTGCGGTATCGCTCAGGCTCACG	Asn 100 to Ala in α chain reverse
TIL1383I-Y101αA-FP	GGCCCTGAACGCCGGCGGTAGCCAG	Tyr 101 to Ala in α chain forward
TIL1383I-Y101αA-RP	ACCAGGCAGTAATAAACTGCGGTATCGCTCAGGCT	Tyr 101 to Ala in α chain reverse
TIL1383I-E101βA-FP	CAGCCCGACCGCCGAAGGCGGCCT	Glu 101 to Ala in β chain forward
TIL1383I-E101βA-RP	ATGGCGCAGAAATAAACGCTTGTCTGGCTGCTGGTGGC	Glu 101 to Ala in β chain reverse
TIL1383I-I106βA-FP	AGGCGGCCTGGCCTTCCCGGGCAA	Ile 106 to Ala in β chain forward
TIL1383I-I106βA-RP	TCTTCGGTCGGGCTGATGGCGCAGAA	Ile 106 to Ala in β chain reverse
TIL1383I-CDR3β Del-FP	CTGATTTTCCCGGGCAACACCATCTAC	Deletion in CDR3β loop forward
TIL1383I-CDR3β Del-RP	TTCGGTCGGGCTGATGGCGCAG	Deletion in CDR3β loop reverse
HLA-A2 R65A-FP	GAGACCGCCAAAGTTAAAGCCCATAGCCAGACC	Arg 65 to Ala in HLA-A2 forward
HLA-A2 R65A-RP	GCCATCCCAGTATTCTGGGC	Arg 65 to Ala in HLA-A2 reverse
