# Supplementary material for: A class-mismatched TCR bypasses MHC restriction via an unorthodox but fully functional binding geometry
Source: Nat Commun. 2022 Nov 23;13:7189. doi: 10.1038/s41467-022-34896-0 (PMC9691722; doi:10.1038/s41467-022-34896-0)
Supplement: Supplementary file 3 — Reporting Summary [file 41467_2022_34896_MOESM3_ESM.pdf]

nature portfolio

Corresponding author(s): Brian Baker

Last updated by author(s): October 31, 2022

Reporting Summary

Nature Portfolio wishes to improve the reproducibility of the work that we publish. This form provides structure for consistency and transparency in reporting. For further information on Nature Portfolio policies, see our Editorial Policies and the Editorial Policy Checklist.

Statistics

For all statistical analyses, confirm that the following items are present in the figure legend, table legend, main text, or Methods section.

n/a	Confirmed	
☐	☒	The exact sample size (*n*) for each experimental group/condition, given as a discrete number and unit of measurement
☐	☒	A statement on whether measurements were taken from distinct samples or whether the same sample was measured repeatedly
☒	☐	The statistical test(s) used AND whether they are one- or two-sided<br>*Only common tests should be described solely by name; describe more complex techniques in the Methods section.*
☒	☐	A description of all covariates tested
☒	☐	A description of any assumptions or corrections, such as tests of normality and adjustment for multiple comparisons
☐	☒	A full description of the statistical parameters including central tendency (e.g. means) or other basic estimates (e.g. regression coefficient) AND variation (e.g. standard deviation) or associated estimates of uncertainty (e.g. confidence intervals)
☒	☐	For null hypothesis testing, the test statistic (e.g. *F*, *t*, *r*) with confidence intervals, effect sizes, degrees of freedom and *P* value noted<br>*Give P values as exact values whenever suitable.*
☒	☐	For Bayesian analysis, information on the choice of priors and Markov chain Monte Carlo settings
☒	☐	For hierarchical and complex designs, identification of the appropriate level for tests and full reporting of outcomes
☒	☐	Estimates of effect sizes (e.g. Cohen's *d*, Pearson's *r*), indicating how they were calculated

Our web collection on statistics for biologists contains articles on many of the points above.

Software and code

Policy information about availability of computer code

Data collection	Biacore control software 2.01 (SPR), NanoTemper PR.ThermControl v2.1 (thermal stability), BD FACSChorus 2.1.1.20.0 (flow)
Data analysis	Biacore T200 evaluation software v3.0 (SPR processing), Biaevaluation 4.1 (SPR fitting), MATLAB 2017a (SPR fitting), OriginPro v2019 (binding, thermal stability), HKL2000 v721 (X-ray), Phaser v2.8.1 (X-ray), PHENIX v1.13-2998 (X-ray), Coot 0.8.9 (X-ray), CNS v2005 (X-ray), Molprobity v4.5 (X-ray), PyMol v2.5 (visualization), Discovery Studio v2019 and v2021 (visualization, electron densities), FlowJo v10.8.1 (flow)

For manuscripts utilizing custom algorithms or software that are central to the research but not yet described in published literature, software must be made available to editors and reviewers. We strongly encourage code deposition in a community repository (e.g. GitHub). See the Nature Portfolio guidelines for submitting code & software for further information.

Data

Policy information about availability of data

All manuscripts must include a data availability statement. This statement should provide the following information, where applicable:

- Accession codes, unique identifiers, or web links for publicly available datasets
- A description of any restrictions on data availability
- For clinical datasets or third party data, please ensure that the statement adheres to our policy

Structural data, including coordinates and structure factors, are available at the Protein Data Bank (https://www.rcsb.org/) under accession code 7RK7 (https://www.wwpdb.org/pdb?id=pdb_00007rk7). Other data are available in the Source data file provided with the manuscript or at the Zenodo data repository at https://zenodo.org/record/7259584.

Field-specific reporting

Please select the one below that is the best fit for your research. If you are not sure, read the appropriate sections before making your selection.

- [x] Life sciences
- [ ] Behavioural & social sciences
- [ ] Ecological, evolutionary & environmental sciences

For a reference copy of the document with all sections, see nature.com/documents/nr-reporting-summary-flat.pdf

Life sciences study design

All studies must disclose on these points even when the disclosure is negative.

Sample size	The study does not involve tests of different experimental samples. For binding experiments, the number of injections and concentrations were selected to ensure coverage across a titration curve as indicated in the figure. The rationale for selection of peptides for validating the specificity analysis was based on testing a large number of those predicted to be positive and a smaller number of those predicted to be negative, with the total balanced by the wells available in plate format. The TIL1383I TCR is an n=1.
Data exclusions	No data were excluded.
Replication	Replicates were performed as indicated in the manuscript (methods, results, and figure legends).
Randomization	As the study involved structural and biophysical studies of only a single TCR and tests of transduced cells, randomization was not performed. For example, in titration experiments, dilutions were made with robotic assistance in a controlled fashion in order of increasing concentration, obviating randomization. Crystal screening was performed similarly by robotics, obviating randomization.
Blinding	As the study involved structural and biophysical studies of only a single TCR, tests of transduced cells, blinding does not apply to the study. For example, in titration experiments, dilutions were made with robotic assistance in a controlled fashion in order of increasing concentration, obviating blinding. Crystal screening was performed similarly by robotics with a need to link conditions to crystals, obviating blinding.

Reporting for specific materials, systems and methods

We require information from authors about some types of materials, experimental systems and methods used in many studies. Here, indicate whether each material, system or method listed is relevant to your study. If you are not sure if a list item applies to your research, read the appropriate section before selecting a response.

Materials & experimental systems

n/a	Involved in the study
[ ]	[x] Antibodies
[ ]	[x] Eukaryotic cell lines
[x]	[ ] Palaeontology and archaeology
[x]	[ ] Animals and other organisms
[x]	[ ] Human research participants
[x]	[ ] Clinical data
[x]	[ ] Dual use research of concern

Methods

n/a	Involved in the study
[x]	[ ] ChIP-seq
[ ]	[x] Flow cytometry
[x]	[ ] MRI-based neuroimaging

Antibodies

Antibodies used	APC/Cyanine7 anti-human CD3 from BioLegend, Catalog No. 300426, Lot No. B277614, used at 10 mg/mL<br>Alexa Fluor® 647 anti-human CD8 Antibody from Biolegend, Cat. # 344726, Lot. # b270007, used at 2.5 mg/mL
Validation	Validation certificates are available from the BioLegend website at https://www.biolegend.com/en-us/certificate-of-analysis by entering in lot numbers below:<br>CD3 antibody Lot# B361145 Expiration date: 03/23/2026<br>CD8 antibody Lot# B270007 Expiration date: 08/31/2023<br>Functional validation of antibodies was via positive and negative controls in the process of T cell transduction and analysis.

Eukaryotic cell lines

Policy information about cell lines

Cell line source(s)	Jurkat 76 were a provided by Dr. Michael Nishimura at Loyola University, originally obtained from ATCC (TIB-152).<br>T2 cells were purchased from ATCC Cat. # crl-1992
Authentication	Authentication of Jurkats and T2 cell lines was performed via functional assays examining IL-2 production by ELISA in the

	presence of exogenous peptide and, for Jurkat cells, cell surface expression of CD3 after TCR transduction.
Mycoplasma contamination	Cells tested negative for mycoplasma.
Commonly misidentified lines (See ICLAC register)	No commonly misidentified cell lines were used.

Flow Cytometry

Plots

Confirm that:

- [x] The axis labels state the marker and fluorochrome used (e.g. CD4-FITC).
- [x] The axis scales are clearly visible. Include numbers along axes only for bottom left plot of group (a 'group' is an analysis of identical markers).
- [x] All plots are contour plots with outliers or pseudocolor plots.
- [x] A numerical value for number of cells or percentage (with statistics) is provided.

Methodology

Sample preparation	Cells were pelleted and resuspended in 100ul of PBS containing 2% Bovine Serum Albumin (PBSA-2%). Antibodies were added as recommended by manufacturer and incubated in the dark at 4C for 25minutes. Cells were then washed two times with 1mL of PBSA-2% and cells resuspended in a final volume of 300uL in PBSA-2%.
Instrument	BD FACS Melody
Software	Software for data collection: BD FACSChorus Software 2.1.1.20.0<br>software for data analysis: FlowJo 10.8.1
Cell population abundance	Flow cytometry was used for analysis only. A total of 100,000 cells were counted and after applying the FSC/SSC gate (singlets) approximately 70% of total cells were used for analysis of CD3 or CD8 expression.
Gating strategy	Gating strategy includes utilizing SSC and FSC gates to identify single cells (singlets). A second set of gates were utilized as quadrants using the SSC and CD3-APC/Cy7 or CD8-AlexaFluor 647 gates to determine positive and negative cells. Untransduced samples served as a negative control for TCR expression. Cells located within the lower right quadrant were considered positive for CD3 or CD8 expression.

- [x] Tick this box to confirm that a figure exemplifying the gating strategy is provided in the Supplementary Information.
